# A Dressing for Fracture

**Published:** 1872-11

**Authors:** B. W. Stone

**Affiliations:** Hopkinsville, Ky.


THE GEORGIA
### MEDICAL COMPANION:
A MONTHLY ADVISER.

VOL. 2. ATLANTA, GA., NOVEMBER, 1872. NO. 11.

### PART I.

### Original Communications and Special Selections.

### A DRESSING FOR FRACTURE.

BY B. W. STONE, M. D., HOPKINSVILLE, KY.

The general surgeon, occasionally, has to deal with cases of fracture, where his dressings are constantly liable to disturbance. This is the case when the subject is an epileptic; or is laboring under any form of delirium. Even an intemperate person may become intoxicated, and remove dressings from a fractured limb. A patient under my care, whilst a resident graduate at the Louisville City Hospital, who had suffered fracture during a spree, became drunk twice, whilst undergoing treatment, and destroyed all the appliances used, making her case a much more serious one.

Without mentioning any other classes, the insane are especially troublesome in this regard. A very large proportion of them will attempt to remove, or at least, disturb any sort of surgical apparatus placed upon them. Even an idiot will, often do so. Especially if the appliances are cumbrous or uncomfortable.

With all such patients as the foregoing, dressings much stronger than the most substantial in ordinary use, should be employed. In fractures of the limbs, I think the follnwing simple plan of dressing, which I have used in this asylum, is deserving of consideration: After the fragments are brought into apposition, I secure them by two layers of the roller with splints of bookbinder's board between, (as ordinarily recommended); outside of this dressing is placed a wooden side-splint, deeply hollowed out on the side next to the limb, which is retained in position by straps of tinned iron or sheet iron; these encircle both the limb and splint, and are securely fastened with screws to the latter. The straps should be from an inch and a half to three inches in width, and should be covered with cloth. In fractures of the forearm, of course, precaution should be taken to keep the two bones separate. The splint also is posterior here.

This dressing is easily applied, the materials are cheaply obtained, it is light enough, is comfortably worn, and is certainly eminently secure against ordinary accident.

I used this plan, with gratifying results, in the case of an insane man, with fracture of the tibia and fibula, who had previously removed three strong, starched bandages. The last one he removed during the eleventh night, after the occurrence of fracture, to accomplish which he had loosed his hands from well-applied leathern straps, which bound them to his bedrails. When observed next day the fragments were entirely ununited. On account of the extensive laceration of tissue, in the neighborhood of the fracture, indicated by the ecchymosis present, a *starched* bandage was used in this case, and a window made in this, through which to observe the condition of the leg, a corresponding inter-space, of course, was left between the overlying straps of metal. The patient recovered, without the slightest shortening of his limb.

Western Lunatic Asylum, of Ky., Sept. 18, 1872.

